# Salinity and temperature influence removal levels of heavy metals and chloride from water by wetland plants

**DOI:** 10.1007/s11356-023-26490-8

**Published:** 2023-03-28

**Authors:** Maria Schück, Maria Greger

**Affiliations:** Department of Ecology, Environment and Plant Sciences, Stockholm University, 106 91, Stockholm, Sweden

**Keywords:** Salinity, Temperature, Wetland plants, Heavy metals, Chloride, Phytodesalination

## Abstract

Stormwater with low temperatures and elevated salinity, common in areas where deicing salt is used, might affect the removal of heavy metals by plants in stormwater treatment systems such as floating treatment wetlands. This short-term study evaluated the effects of combinations of temperature (5, 15, and 25 °C) and salinity (0, 100, and 1000 mg NaCl L^−1^) on the removal of Cd, Cu, Pb, and Zn (1.2, 68.5, 78.4, and 559 μg L^−1^) and Cl^−^ (0, 60, and 600 mg Cl^−^ L^−1^) by *Carex pseudocyperus*, *C. riparia*, and *Phalaris arundinacea.* These species had previously been identified as suitable candidates for floating treatment wetland applications. The study found high removal capacity in all treatment combinations, especially for Pb and Cu. However, low temperatures decreased the removal of all heavy metals, and increased salinity decreased the removal of Cd and Pb but had no effect on the removal of Zn or Cu. No interactions were found between the effects of salinity and of temperature. *Carex pseudocyperus* best removed Cu and Pb, whereas *P. arundinacea* best removed Cd, Zu, and Cl^−^. The removal efficacy for metals was generally high, with elevated salinity and low temperatures having small impacts. The findings indicate that efficient heavy metal removal can also be expected in cold saline waters if the right plant species are used.

## Introduction

Stormwater commonly contains elevated levels of heavy metals, especially Cd, Cu, Pb, and Zn, which may reach harmful concentrations. Additionally, water temperature and salinity levels in stormwater vary over the year and between locations. The temperature of stormwater follows the air temperature (Van de Moortel et al. 2010), resulting in high temperatures in warm weather, but it can also reach sub-zero temperatures under winter conditions. The combination of low temperature and elevated salinity levels is commonly found in winter when deicing salt, commonly NaCl, is used. Stormwater originating from areas where deicing salt is not used will reach sub-zero temperatures in winter while maintaining low salinity. In summer and in locations with warmer climates throughout the year, stormwater is generally warm and has low salinity as deicing salt is not used. However, warm and salty conditions may occur in seaside stormwater treatment systems (Sanicola et al. 2019; Szota et al. 2015).

Variation in either temperature or salinity may affect plant-based stormwater treatment systems, such as constructed wetlands and floating wetlands, which can be used to reduce heavy metal and chloride concentrations in stormwater.

Temperature affects the metabolism and membrane function of plants, profoundly affecting plant growth and survival, key factors for the success of plant-based water treatment systems. It also directly affects metal absorption and translocation from roots to shoots (Brunham and Bendell 2011), generally resulting in higher accumulation at higher temperatures. Since plant adsorption and plant-mediated sedimentation are physical processes, they are less influenced by temperature than are absorption and translocation. In addition, temperature affects the amount and bioavailability of metals in the water by affecting the release of heavy metals from sediment (Li et al. 2013) and the speciation into more or less bioavailable complexes (Poot et al. 2007).

Salinity affects the osmotic balance of plants and can be cytotoxic, influencing the metabolism and uptake of water and ions, including heavy metal ions and chloride. This results in secondary effects on growth, hormone levels, and ultimately survival. Salt-tolerant plants, known as halophytes, maintain growth at high salinity levels; they may also be especially efficient for heavy metal removal due to shared tolerance mechanisms for both stressors (Lutts and Lefèvre 2015; Manousaki and Kalogerakis 2011). Moreover, salinity has been found to both increase and decrease the bioavailability of metals (Greger et al. 1995). Sodium promotes the desorption of metal ions bound to soil and sediment particles (Dołęgowska et al. 2022; Du Laing et al. 2008; Greger et al. 1995). Chloride promotes the formation of soluble metal-Cl complexes, increasing the metal bioavailability, or less soluble metal-Cl complexes, lowering the metal bioavailability (Dołęgowska et al. 2022). The combined effect of salinity’s often positive effects on bioavailability and negative effects on plant health results in both increased and decreased plant uptake of heavy metals (Dołęgowska et al. 2022; Du Laing et al. 2008; Fritioff et al. 2005; Greger et al. 1995; Han et al. 2012a; Szota et al. 2015; Zhou et al. 2019). The particular result depends on the plant species, substrate characteristics, and concentration of NaCl and metals.

The effects on metal uptake of salinity and temperature stress vary between plant species, as recorded in several studies (Brunham and Bendell 2011; Fritioff et al. 2005). Furthermore, interactions between salinity and temperature impacts on heavy metal uptake have been recorded in submersed plants (Fritioff et al. 2005) and macroalgae (Bastos et al. 2019). Such interactions may result from several types of mechanisms. The combined stress of two abiotic stressors, such as salt and low temperature, may have synergetic effects on plants and result in more severe effects than when stressors occur singly (Mittler and Blumwald 2010). On the other hand, salinity and temperature have opposite effects on transpiration, which affects the passive influx of ions such as heavy metal ions into the root apoplast (Lutts and Lefèvre 2015). Salinity decreases the leaf expansion rate and thus lowers transpiration (Ebrahimi and Bhatla 2011), whereas heat increases transpiration. The combined effect of salinity and temperature stress is thus difficult to predict.

Identifying how salinity and temperature affect plant species’ metal and chloride removal capacity is integral to predicting the pollution removal potential of plant-based treatment systems. It is also useful for further optimizing the methods to mitigate the potential negative effects of expected environmental conditions by adjusting the treatment system size and plant composition. By determining the effects in a controlled small-scale setting, potentially costly mistakes in situ can be avoided.

To further understand the potential of plants for stormwater remediation, this study evaluated the effects of salinity and temperature on the uptake capacities for Cd, Cu, Pb, Zn, and Cl^−^ of *Carex pseudocyperus* L., *C. riparia* Curtis, and *Phalaris arundinacea* L., using a multifactorial approach. These species were found to be the most efficient removers of Cd, Cu, Pb, and Zn, and low levels of Cl^−^ at 17 °C in a previous comparative study of 34 wetland plant species (Schück and Greger 2020, 2022). Our hypotheses were (1) increased temperature increases pollutant removal; (2) increased salinity decreases pollutant removal from water; and (3) the response to increased temperature and salinity differs between species.

## Materials and methods

### Growth conditions

The experiments were conducted in three identical greenhouse chambers (16 h light day^−1^) located at Stockholm University (N 59° 36′, E 18° 06′) during the winter months, January–March, of 2020. The greenhouse chambers had three different temperature settings: 5, 15, and 25 °C. The first two temperatures were based on average temperatures in spring and summer in central Sweden, whereas 25 °C can be expected in the air and water at sunny locations in summer (Persson 2015). As the 5 °C greenhouse chamber was chilled with outside air, the average daily temperature varied between 4.1 and 9.6 °C with an average of 6.4 °C due to the unexpectedly warm winter weather. The RH was 55% in the 15 and 25 °C greenhouse chambers and 75% in the 5 °C greenhouse chamber.

### Plant material

Plants were collected from the field in Flemingsberg and Stockholm (*C. riparia* and *C. pseudocyperus*) or purchased from Vegtech AB (*P. arundinacea*). After being thorough cleaned of soil and debris, the plants were grown in 25% modified Hoagland solution (Eliasson 1978) with the addition of 1 mM K_2_SiO_3_ (pH adjusted to 5.9 ± 0.1 with NaOH) in the 15 °C greenhouse chamber described above. New plants were propagated from the original plants by cutting tiller clumps of *C. riparia* and *C*. *pseudocyperus* and by stem cuttings from *P. arundinacea*. The experiment began approximately 3 months after propagation.

### Experimental setup

Specimens of *C. riparia*, *C. pseudocyperus*, and *P. arundinacea* of similar size and shape were selected for the test (Table 1). First, the plants were acclimatized to the conditions in each greenhouse chamber. Plants of each species were randomly divided into three groups, one for each greenhouse chamber. Each plant was attached to a cellular polyethylene plate to keep it floating and then placed in a 10-L container filled with 7.5 L of aerated 1% modified Hoagland (Eliasson 1978) and 0.1 mM K_2_SiO_3_ solution, based on typical N, P, and Si levels in stormwater (Alm et al. 2010; Billberger 2011). The ratio between root biomass and solution volume in this study was based on the field trial of Borne et al. (2014), with both experiments having approximately 1 g root dry weight (DW) L^−1^.

**Table 1:** Treatment averages (± SE) of fresh and dry weights, and ratios of the dry:fresh weight and root:shoot weight; letters indicate differences between species (*p* < .05); *n* = 45

Species	Fresh weight, start (g)	Dry weight, end (g)	Dry:fresh weight ratio, end	Root:shoot ratio, end
*Carex pseudocyperus*	29.22 ± 0.67^b^	5.44 ± 0.19^b^	0.186 ± 0.004^c^	0.308 ± 0.016^a^
*Carex riparia*	27.76 ± 0.61^b^	6.98 ± 0.19^a^	0.252 ± 0.004^a^	0.209 ± 0.010^b^
*Phalaris arundinacea*	31.73 ± 0.68^a^	7.03 ± 0.25^a^	0.222 ± 0.006^b^	0.191 ± 0.011^b^

After 12 days of acclimatization to the different temperatures, the solution in each container was replaced with 7.5 L of 1% modified Hoagland (Eliasson 1978) and 0.1 mM K_2_SiO_3_ solution, with 1.2 μg L^−1^ Cd, 68.5 μg L^−1^ Cu, 78.4 μg L^−1^ Pb, and 559 μg L^−1^ Zn added as CdCl_2_, CuCl_2_, PbCl_2_, and ZnCl_2_. Sodium chloride (NaCl) was added to two-thirds of the containers, which together with the Cl^−^ originating from the metal salts and the nutrient solution, yielded chloride concentrations of < 1, 60, and 600 mg Cl^−^ L^−1^ (< 1, 1.7, and 17 mM Cl), respectively. This resulted in nine different combinations of temperature and salinity, with five replicates per species. The heavy metal and salinity levels used in the experiment were based on levels measured in stormwater ponds in areas where deicing salt was used at various times of the year (Alm et al. 2010; Billberger 2011; Semadeni-Davies 2006). Containers without plants but otherwise identical were placed alongside the other treatments in each greenhouse chamber to account for evaporation and the adsorption of metals to the containers. Solution samples were taken after 0 and 120 h of exposure, filtered through 0.45-μm single-use filters, and stored at 3 °C before analysis. The experiment ended after 5 days of exposure, based on the recommended average hydraulic retention time in stormwater ponds (Seffel 2015). The plants were weighed, rinsed three times in deionized water, divided into roots and shoots, and dried for 48 h at 70 °C.

### Analytical methods

Heavy metal concentrations were analyzed with flame and furnace atomic absorption spectrometry (using Varian 50B and Agilent 240AA devices). Chloride concentrations were analyzed with ion chromatography according to ISO 10304–1:2007 using the IonPac AG9-HC column, Hamilton PRP X-100 79,455 ion-exchange column, Shimadzu CDD-10A VP conductivity meter, and Shimadzu LabSolutions, version 5.90. The effects on Cl^−^ removal of species, temperature, and salinity level were only evaluated for salinity levels of 60 and 600 mg L^−1^, since the < 1 mg L^−1^ treatment was considered not relevant to the scope of the study, as in it, the plants were exposed to very low chloride concentrations.

### Calculation and statistical methods

The metal removal capacity by the plants was calculated as:

1 $$\mathrm{Metal\;removal\;capacity\;per\;plant\;}(\mathrm{\% \;Me\;}{\mathrm{plant}}^{-1}) = (1 - ({[\mathrm{Me}]}_{\mathrm{t}120}\times {V}_{\mathrm{t}120})/({[\mathrm{Me}]}_{\mathrm{t}120\mathrm{c}} \times {V}_{\mathrm{t}120\mathrm{c}})) \times 100$$

where [Me]_t120_ is the metal concentration and *V*_t120_ is the volume of solution remaining in each container after 120 h of exposure, and [Me]_t120c_ and *V*_t120c_ are the metal concentration and solution volume, respectively, in the no-plant control containers after 120 h of exposure.

As the Cl^−^ starting concentration differed between treatments, the removal capacity was calculated in absolute terms for easier comparison between treatment levels:

2 $$\mathrm{Chloride\;removal\;capacity\;per\;plant\;}(\mathrm{mg\;}{\mathrm{Cl}}^{-} {\mathrm{\;plant}}^{-1}) = ({[\mathrm{Cl}-]}_{\mathrm{t}120\mathrm{c}} \times {V}_{\mathrm{t}120\mathrm{c}}) - ({[{\mathrm{Cl}}^{-}]}_{\mathrm{t}120} \times {V}_{\mathrm{t}120})$$

where [Cl^−^]_t120_ is the chloride concentration and *V*_t120_ is the volume of solution remaining in each container after 120 h of exposure, and [Cl^−^]_t120c_ and *V*_t120c_ are the chloride concentration and solution volume, respectively, in the no-plant control after 120 h of exposure.

The metal and chloride removal capacities were also calculated per biomass (DW) to even out the small differences in plant size (Table 1) between treatments:

3 $$\mathrm{Metal\;removal\;capacity\;per\;biomass\;}(\mathrm{\%\;removed\;Me\;g\;}{\mathrm{DW}}^{-1}) =\mathrm{ Metal\;removal\;capacity\;}(\mathrm{\%\;Me\;}{\mathrm{\;plant}}^{-1})/\mathrm{g\;}{\mathrm{DW}}^{-1}$$

4 $$\mathrm{Chloride\;removal\;capacity\;per\;biomass\;}(\mathrm{mg\;removed\;}{\mathrm{Cl}}^{-} {\mathrm{\;DW}}^{-1}) =\mathrm{\;Chloride\;removal\;capacity\;}(\mathrm{mg\;}{\mathrm{Cl}}^{-} {\mathrm{\;plant}}^{-1})/\mathrm{g\;}{\mathrm{\;DW}}^{-1}$$

All data were analyzed with R, version 3.6.3. All response variables were checked for normality with the Shapiro–Wilk test and homogeneity with Levene’s test. They were then analyzed with three-way ANOVAs with species, temperature, and salinity as factors. Significant differences between means of each treatment were identified using the Tukey HSD test (*p* < 0.05).

## Results

### Growth and plant health

**Fig. 1:**
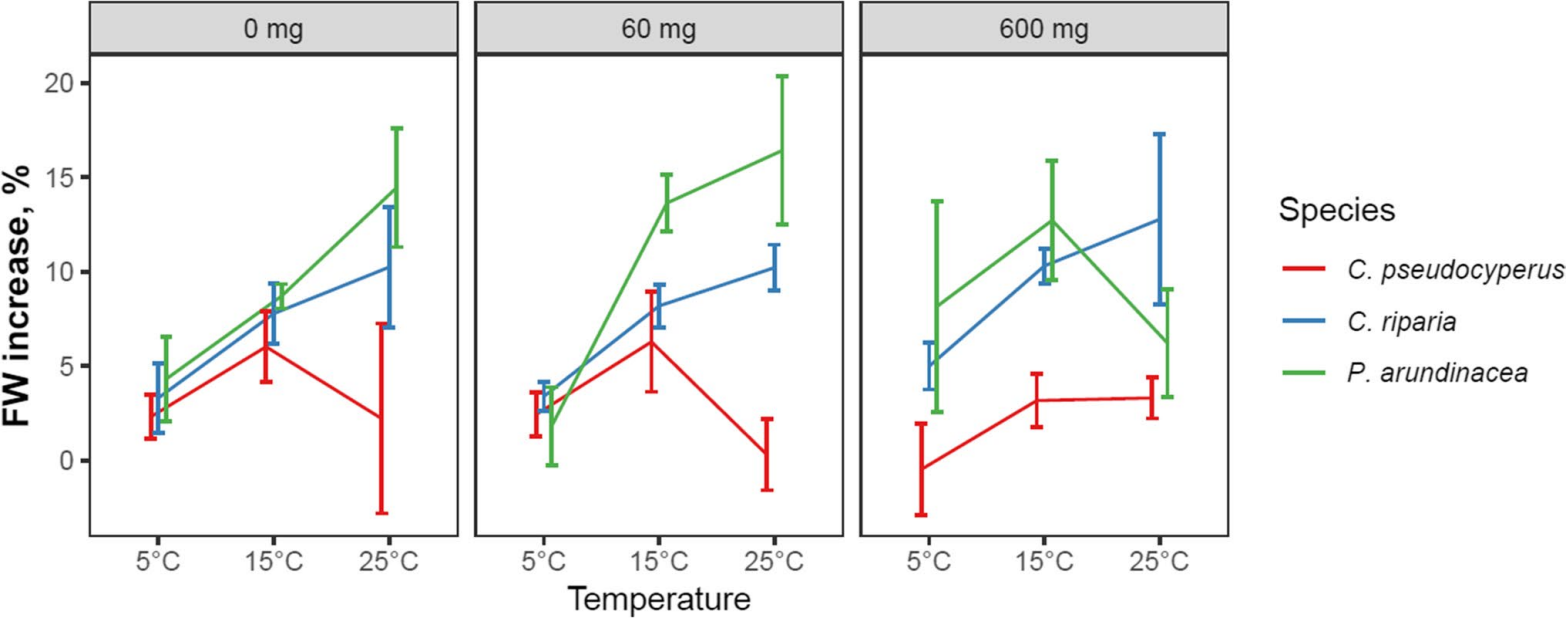
Effects of species, temperature, and salinity on heavy metal removal on fresh weight increase (± SE) after 5 days of exposure; *n* = 5

Temperature and species affected the growth of the plants, measured as the increase in fresh weight, independent of each other (Table 2 and Fig. 1). The higher temperatures, 15 and 25 °C, resulted in between 1.4 and 3.6 times more growth than did the 5 °C temperature (Fig. 1). *Carex riparia* and *P. arundinacea* grew between 1.7 and 6.4 times more than did *C. pseudocyperus*. Salinity did not affect plant growth. All plants remained visibly healthy (e.g., no dieback or yellowing of leaves) throughout the experiment (not shown).

**Table 2:** Main and interaction effects of species, temperature, and salinity on fresh weight increase (%) after 5 days of exposure to various temperatures and salinity concentrations (% g DW^−1^). Values in bold indicate significance according to three-way ANOVA analysis (*p* < .05); *Df*, degrees of freedom

	Df	*p* value
Species	2	** < .001**
Salinity	2	.955
Temperature	2	** < .001**
Species:salinity	4	.634
Species:temperature	4	.102
Salinity:temperature	4	.723
Species:salinity:temperature	4	.124
Residuals	108

### Metal removal

The results of the factorial test indicate that species and temperature affected the removal of all four metals (Table 3). Salinity affected the removal of Pb and Cd but not that of Cu and Zn. Interaction effects were seen between species and temperature in Cd and Zn removal and between salinity and species in Cd removal.

**Table 3:** Main and interaction effects of species, temperature, and salinity on removal of Cd, Cu, Pb, and Zn (% g DW^−1^). Values in bold indicate significance according to three-way ANOVA analysis (*p* < .05); *Df*, degrees of freedom

	Df	Cd	Cu	Pb	Zn
Species	2	** < .001**	** < .001**	** < .001**	** < .001**
Salinity	2	** < .001**	.263	** < .001**	.313
Temperature	2	** < .001**	**.003**	**.003**	** < .001**
Species:salinity	4	**.048**	.949	.480	.291
Species:temperature	4	**.028**	.073	.510	**.021**
Salinity:temperature	4	.865	.370	.680	.731
Species:salinity:temperature	8	.782	.678	.469	.615
Residuals	107				

**Fig. 2:**
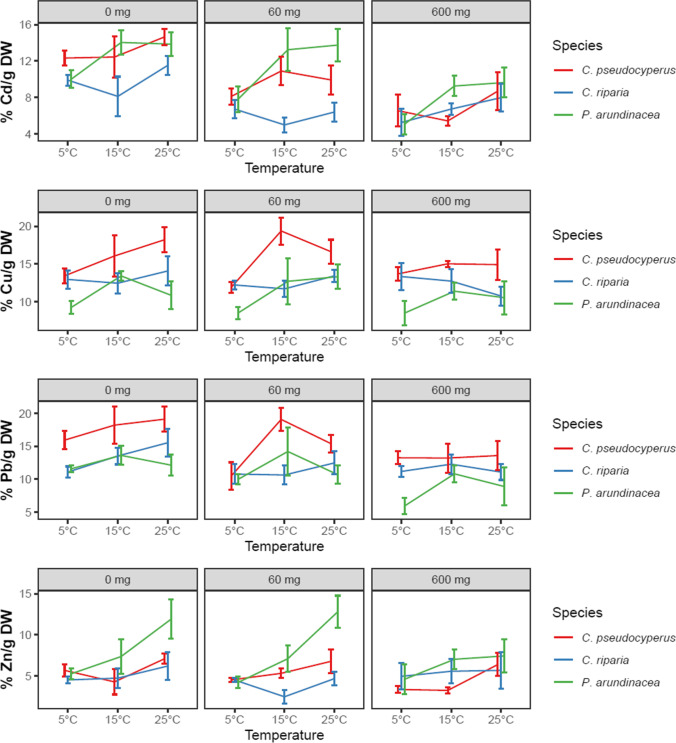
Effects of species, temperature, and salinity on heavy metal removal capacity per biomass (% removed Me g^−1^ plant DW ± SE) after 5 days of exposure; initial metal concentrations were 1.2 μg Cd L^−1^, 68.5 μg Cu L^−1^, 78.4 μg Pb L^−1^, and 559 μg Zn L^−^.^1^; *n* = 5

Increasing temperature had a positive effect on the metal removal capacity of the plants (Fig. 2 and Table 3). Removal of Cu and Pb was up to 63 and 82% higher at 15 and 25 °C than at 5 °C. For Cd, the removal capacities of *C. pseudocyperus* and *P. arundinacea* were up to 34 and 90% higher at 15 and 25 °C than at 5 °C. The Cd removal capacity of *C. riparia* remained low at all temperatures. At 5 °C, all species had similar Cd removal capacities. For Zn, the removal was up to 38 and 91% higher at 25 °C than at lower temperatures in *C. pseudocyperus* and *C. riparia*, respectively, but the effect was stronger in *P. arundinacea* than in the other species as it had up to 205% higher removal at 25 °C compared to 5 °C. In *P. arundinacea*, the Zn uptake was not as high at 15 °C as at 25 °C, but it was still between 29 and 189% higher than in the other species at that temperature; at 5 °C, all species had similar Zn removal capacities.

Increasing salinity harmed the Cd and Pb removal capacities of the plants (Fig. 2 and Table 3). For Cd, the *Carex* species *C. pseudocyperus*, and *C. riparia* removed up to 129% and 88% more Cd, respectively, at < 1 mg Cl^−^ L^−1^ than at 60 or 600 mg Cl^−^ L^−1^. In *P. arundinacea*, on the other hand, Cd removal capacity remained high at 60 mg Cl^−^ L^−1^, decreasing only at 600 mg Cl^−^ L^−1^; high salinity decreased Pb removal, which was between 21 and 49% lower at 600 mg Cl^−^ L^−1^ than at < 1 mg Cl^−^ L^−1^. No effects of salinity on the removal capacities for Cu or Zn were seen in all three plant species.

The species differed in their removal capacities for all metals (Fig. 2 and Table 3). For Cu and Pb, *C. pseudocyperus* removed up to 68 and 124% more metal from the solution than did *C. riparia* or *P. arundinacea*. For Cd and Zn, interactions between species, on one hand, and salinity or temperature, on the other, resulted in the more complex performance of the species under the different conditions described above. However, *P. arundinacea* and *C. pseudocyperus* achieved the highest removal of Cd at < 1 and 60 mg Cl^−^ L^−1^ at 15 and 25 °C. *P. arundinacea* achieved the highest Zn removal at < 1 and 60 mg Cl^−^ L^−1^ at 25 °C.

### Chloride removal

**Fig. 3:**
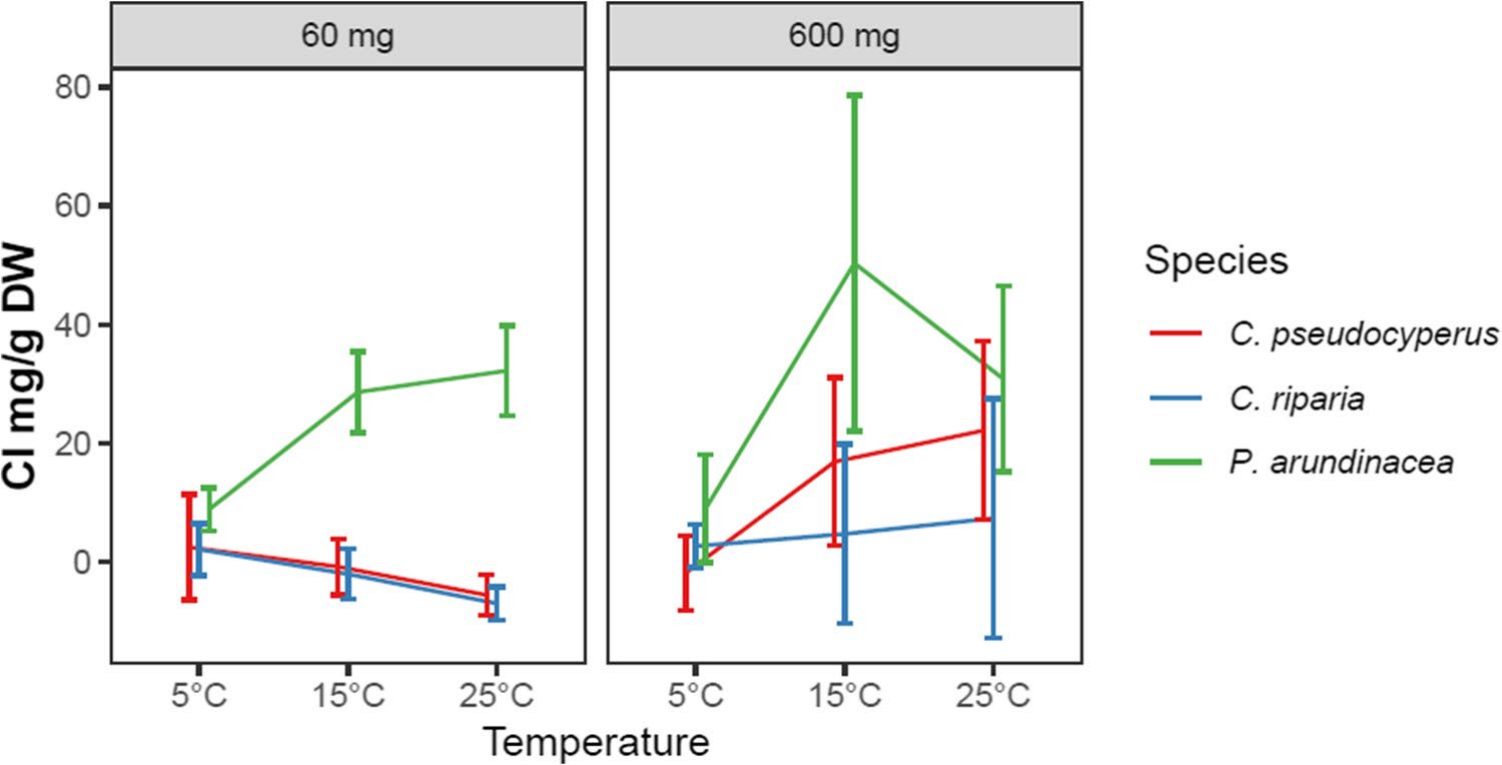
Effects of species, temperature, and salinity on chloride removal capacity per biomass (mg removed Cl^−^ g^−1^ plant DW ± SE) after 5 days of exposure; initial chloride concentrations were 60 and 600 mg Cl^−^ L^−1^; *n* = 5. Note that some standard errors are higher than the mean values due to specimens of the same treatment both releasing and accumulating chloride

Neither temperature nor salinity affected the chloride removal capacity of the plants (Table 4). The only difference in chloride removal capacity was seen between species, with *P. arundinacea* showing up to 30 times higher removal capacity than that of the *Carex* species (Table 4 and Fig. 3).

**Table 4:** Main and interaction effects of species, temperature, and salinity on removal of Cl^−^ (mg g DW^−1^). Values in bold indicate significance according to three-way ANOVA analysis (*p* < .05); *Df*, degrees of freedom

	Df	*p* value
Species	2	** < .001**
Salinity	1	.101
Temperature	2	.172
Species:salinity	2	.853
Species:temperature	4	.386
Salinity:temperature	2	.415
Species:salinity:temperature	4	.793
Residuals	72

### Overall removal efficacy

The overall removal capacity differed between treatments and between the heavy metals and chloride (Figs. 2 and 3). Plants removed up to 95% of the Cu and Pb from the water, the average removal across all treatments being 80 and 77% for Cu and Pb, respectively (Table 5). Plants removed 80% of the Zn and Cd in a few treatments, but the removal was generally lower than for Cu or Pb. Chloride, present in much higher concentrations than the metals, decreased less. The concentration decrease in pots with no plants after 120 h of exposure was 7.3, 6.3, 19.9, 1.5, and 1.3% of the initial concentration for Cd, Cu, Pb, Zn, and Cl^−^, respectively, and was unaffected by salinity or temperature (not shown).

**Table 5:** Uptake capacity per plant biomass (μg Me g biomass DW^−1^ ± SE and mg Cl^−^ g biomass DW^−1^ ± SE) and removal capacity per whole plant (% plant^−1^) for each treatment after five days of exposure; initial concentrations of the solution were 1.2 μg Cd L^−1^, 68.5 μg Cu L^−1^, 78.4 μg Pb L^−1^, 559 μg Zn L^−1^, and 60 and 600 mg Cl^−^ L^−1^; *n* = 5

Species	Temperature	Salinity	Cd		Cu		Pb		Zn		Cl^−^	
	°C	mg Cl^−^ L^−1^	μg g DW^−1^	%	μg g DW^−1^	%	μg g DW^−1^	%	μg g DW^−1^	%	mg g DW^−1^	%
*Carex pseudocyperus*	5	0	0.4 ± 0.1	42 ± 7	73 ± 10	67 ± 4	70 ± 8	85 ± 2	262 ± 31	30 ± 3	-	-
		60	0.7 ± 0.3	54 ± 5	74 ± 13	70 ± 3	10 ± 3	58 ± 8	215 ± 18	26 ± 3	2 ± 9	0 ± 11
		600	0.4 ± 0.0	44 ± 11	60 ± 7	74 ± 3	24 ± 11	71 ± 3	156 ± 21	18 ± 3	− 2 ± 6	− 2 ± 1
	15	0	0.3 ± 0.1	53 ± 10	102 ± 18	86 ± 9	59 ± 4	97 ± 2	196 ± 73	25 ± 9	-	-
		60	0.6 ± 0.1	65 ± 4	112 ± 28	91 ± 2	29 ± 3	90 ± 3	216 ± 17	25 ± 2	− 1 ± 5	− 4 ± 7
		600	0.3 ± 0.1	22 ± 12	77 ± 11	85 ± 2	24 ± 7	73 ± 10	143 ± 17	19 ± 3	17 ± 14	0 ± 2
	25	0	0.9 ± 0.1	78 ± 7	96 ± 12	85 ± 2	84 ± 13	90 ± 6	285 ± 24	34 ± 5	-	-
		60	1.1 ± 0.3	82 ± 6	84 ± 13	87 ± 3	30 ± 4	82 ± 8	232 ± 55	37 ± 9	− 6 ± 3	− 7 ± 4
		600	0.6 ± 0.2	43 ± 8	66 ± 4	796	34 ± 11	72 ± 7	269 ± 52	35 ± 9	22 ± 15	2 ± 2
*Carex riparia*	5	0	0.4 ± 0.1	49 ± 8	67 ± 8	90 ± 2	44 ± 11	82 ± 5	207 ± 21	33 ± 2	-	-
		60	0.3 ± 0.1	31 ± 13	81 ± 18	92 ± 2	13 ± 4	78 ± 8	213 ± 14	33 ± 3	2 ± 4	1 ± 9
		600	0.4 ± 0.1	59 ± 10	56 ± 11	94 ± 5	20 ± 3	82 ± 9	177 ± 44	34 ± 11	3 ± 4	− 1 ± 1
	15	0	0.2 ± 0.1	50 ± 11 ±	67 ± 7	87 ± 2	45 ± 11	95 ± 1	178 ± 38	32 ± 7	-	-
		60	0.3 ± 0.1	25 ± 8	56 ± 6	77 ± 3	18 ± 4	70 ± 1	94 ± 26	16 ± 5	− 2 ± 4	− 7 ± 8
		600	0.2 ± 0.1	24 ± 10	58 ± 11	88 ± 8	18 ± 4	84 ± 7	208 ± 49	41 ± 13	5 ± 15	0 ± 3
	25	0	0.5 ± 0.1	54 ± 6	73 ± 9	81 ± 3	61 ± 11	89 ± 3	265 ± 77	34 ± 7	-	-
		60	0.6 ± 0.2	50 ± 9	86 ± 9	85 ± 4	16 ± 4	78 ± 9	189 ± 34	31 ± 8	− 7 ± 3	− 14 ± 5
		600	0.2 ± 0.1	11 ± 4	58 ± 10	73 ± 9	25 ± 8	75 ± 6	252 ± 81	38 ± 14	7 ± 20	0 ± 3
*Phalaris arundinacea*	5	0	0.6 ± 0.1	61 ± 4	52 ± 8	65 ± 6	44 ± 8	73 ± 4	281 ± 47	33 ± 4	-	-
		60	0.5 ± 0.1	58 ± 9	52 ± 6	69 ± 6	12 ± 2	76 ± 6	181 ± 22	31 ± 4	9 ± 4	15 ± 7
		600	0.4 ± 0.1	56 ± 13	48 ± 11	66 ± 7	8 ± 1	45 ± 10	220 ± 70	33 ± 12	9 ± 9	0 ± 2
	15	0	0.5 ± 0.1	63 ± 6	87 ± 7	82 ± 4	52 ± 17	83 ± 6	296 ± 82	43 ± 12	-	-
		60	0.0 ± 0.1	6 ± 12	61 ± 12	70 ± 4	20 ± 4	78 ± 7	272 ± 52	39 ± 2	29 ± 7	44 ± 9
		600	0.2 ± 0.1	21 ± 11	62 ± 10	80 ± 5	26 ± 7	76 ± 7	310 ± 51	51 ± 11	50 ± 28	7 ± 5
	25	0	0.6 ± 0.1	83 ± 8	55 ± 9	75 ± 9	44 ± 6	85 ± 8	468 ± 100	82 ± 11	-	-
		60	0.6 ± 0.1	81 ± 7	94 ± 29	87 ± 2	23 ± 5	70 ± 5	499 ± 109	83 ± 9	32 ± 8	48 ± 13
		600	0.6 ± 0.1	66 ± 7	50 ± 12	70 ± 9	16 ± 6	57 ± 14	307 ± 82	50 ± 10	31 ± 16	3 ± 2

## Discussion

This work shows that wetland plants can decrease the concentrations of heavy metals and chloride in water and that the removal of metals, but not chloride, is influenced by temperature, and, for Cd and Pb, also by salinity (Tables 3 and 4 and Figs. 2 and 3). The extent of removal also depends on plant species and substance. Contrary to several other studies, we did not see interaction effects between salinity and temperature on removal capacity.

In line with our first hypothesis, we confirmed that higher temperatures result in higher removal of all heavy metals, independent of salinity (Table 3). Removal of these metals was approximately 15–55% lower at 5 °C than in treatments at 15 and 25 °C (Fig. 2).

Higher temperatures increase the metabolic activity of plants by stimulating cambial activity and increase photosynthesis, resulting in increased growth and transpiration (Qaderi et al. 2019). This results in increased accumulation of ions, including metals, which follow the water stream into the roots (He et al. 2023; Lutts and Lefèvre 2015; Rabêlo et al. 2021). In our study, growth and the uptake of Cu and Pb in all species, and the uptake of Cd and Zn in *P. arundinacea*, followed the same pattern, with higher uptake and growth at 15 and 25 °C than at 5 °C (Figs. 1 and 2).

Low temperatures can affect the uptake of substances in different ways, as temperature alters membrane selectivity and the available amount of energy for transport against a concentration gradient (He et al. 2023; White 2011). This has a greater effect on substances mainly transported by symplastic transport, whereas substances mainly transported by apoplastic transport are less affected. This might explain the differences between uptakes of Cu and Pb, which decreased only at 5 °C, and Zn, which decreased at both 5 and 15 °C, versus at 25 °C (Fig. 2). Zinc uptake is tightly regulated, mainly mediated by ZIP transporters, which decrease uptake at low temperatures as metabolism slows (Hart et al. 1998; Küpper and Andresen 2016). Removal of Cd displays the same temperature effect pattern as does Cu and Pb removal in *P. arundinacea*, but was not affected by temperature in *Carex* spp. (Fig. 2). Plant species differ in their sensitivity to low temperatures, which might explain the differences in Cd and Zn removal between *P. arundinacea* and the *Carex* species. These species differences might be attributed to differences between species in the ability to release oxygen from the roots at low temperatures (Allen et al. 2002), in shoot demand, and in membrane composition (White 2011), all resulting in differences in ion uptake.

High temperature did not increase Cl^−^ removal (Table 4 and Fig. 3), unlike in non-wetland plants, in which Cl^−^ accumulation increase with increased temperature under waterlogged conditions due to increased hypoxia that decreases the metabolic activity of plants, including Cl^−^ exclusion mechanisms (George et al. 2011; West and Taylor 1980; Wu and Li 2019). Instead, as the evaluated plants can release oxygen from their roots into the rhizosphere, adapting to waterlogged conditions (Moog and Brüggemann 1998), they can maintain the high metabolic activity necessary to restrict Cl^−^ accumulation (Wu and Li 2019).

The effects of temperature on pollutant uptake were generally small, except in the case of Zn removal by *P. arundinacea*, which increased greatly at 25 °C (Fig. 2). Accordingly, we suggest that *P. arundinacea* should be used in conditions where temperatures of 25 °C and above can be expected, to utilize its ability to remove Zn.

Our second hypothesis, i.e., that increased salinity would decrease pollutant removal, was confirmed for Cd and Pb, independent of temperature (Table 3). Removal of these metals was approximately 40% lower at 600 mg Cl^−^ L^−1^ than in treatments with < 1 mg Cl^−^ L^−1^ (Fig. 2).

High salinity may have decreased uptake via several mechanisms. Exposure to NaCl stimulates the lignification of root epidermis, creating a physical barrier that reduces heavy metal uptake (Cheng et al. 2012). Salinity causes enhanced expression of antiporters NHX1 (Na^+^/H^+^ antiporter) and CaCA (Ca^2+^/cation antiporter superfamily) in plants which promotes Cd tolerance (Wang et al. 2020; Zheng et al. 2021). Additionally, decreased Cd bioavailability due to the formation of Cd-Cl^−^ complexes can explain the reduced Cd uptake at increased salinity levels (Greger et al. 1995; Mei et al. 2014). We saw a clear decrease in Pb accumulation with increasing salinity (Fig. 2). These results match those observed in earlier studies, especially in hydroponic conditions (Cheng et al. 2012; Liang et al. 2019). Such decreased uptake at high salinity has been explained by lower availability (Costa et al. 2020), competition between uptake sites (Bond et al. 1988), and increased exclusion capacity (Mahon and Carman 2008). The uptake of Cu and Zn was unaffected by salinity (Table 3). High salinity often increases metal uptake in plants rooted in soil or sediment, as Na promotes heavy metal uptake ability by binding to particles, thus remobilizing heavy metals by ion exchange (Greger et al. 1995; Liu et al. 2019). We saw none of these positive salinity effects, likely since the metals were readily available at all salinities, as we used hydroponic culture instead of soil. The Cl^−^ uptake was unaffected by salinity (Fig. 2), corroborating Cram’s (1983) finding of only minor differences in tissue concentration during the first week of exposure to a Cl-rich solution.

Salinity can alleviate growth reduction in plants simultaneously exposed to toxic levels of heavy metals, thereby increasing the plant uptake of metals (Han et al. 2012b; Wang and Song 2019; Zhou et al. 2019). These studies suggest that salinity causes this effect by triggering synthesis of high levels of antioxidants, which also protects the plants from heavy metal stress which otherwise reduces growth. Salinity-increased growth was not the case in this study (Table 2), likely since our metal concentrations were lower. More common is decreased growth due to the negative effects of salinity on plant metabolism by decreased osmotic potential and chloride ion toxicity, resulting in decreased on transpiration and ion accumulation (Ebrahimi and Bhatla 2011; Munns and Tester 2008; Schück and Greger 2022). Salinity-decreased growth was also not found here (Table 2). However, we only measured the weight of the whole plant, so small weight changes caused by decreased leaf expansion or root tip growth, common responses to salinity stress (Munns and Tester 2008), may not have been detected.

Decreased heavy metal removal under saline conditions has earlier been found in biofilters, constructed wetlands, and floating wetlands (Huang et al. 2017; Liang et al. 2019; Szota et al. 2015). This indicates a wider problem with heavy metal removal in plant-based systems under saline conditions, which may limit their usefulness under such circumstances.

The third hypothesis, i.e., that the response to increased temperature and salinity differs between species, was confirmed for all metals, chloride, and growth (Tables 2, 3, and 4). *Carex pseudocyperus* better removed Cu and Pb, correlated with its higher root:shoot ratio (Table 1 and Fig. 2). As these metals are mainly restricted to belowground biomass (Jamla et al. 2021; Küpper and Andresen 2016), a higher proportion of roots than shoots is beneficial. The halophyte *P. arundinacea* was the most efficient remover of Cd, Cl^−^, and Zn, and its Cd removal was less decreased by salinity than in the other two species, which are glycophytes. The salinity tolerance differs between species (Schück and Greger 2022), with salt-tolerant plants having been shown to more efficiently detoxify ROS to prevent oxidative damage, which is caused by salinity and by toxic concentrations of heavy metals (Lutts and Lefèvre 2015). However, in our study, oxidative stress caused by heavy metals was unlikely due to low metal concentrations. Other mechanisms of salinity tolerance are differences in the ability to synthesize osmoprotectants and antioxidants, and to control the influx, transport, compartmentalization, and efflux of Cl^−^, Na, and heavy metals, which contribute to differences between species in stress tolerance against salinity, temperature, and heavy metals (Jamla et al. 2021; Lutts and Lefèvre 2015).

## Conclusion

This study demonstrates that temperature and salinity affect the removal of heavy metals by wetland plants. Elevated temperature increases the heavy metal removal, indicating that a higher removal capacity can be expected in plants year-round in a warm climate and in late spring, summer, and early autumn rather than in cold winter months in a cold climate. Elevated water salinity decreases the Cd and Pb removal by plants, indicating that deicing salt or the presence of seawater will likely lower removal efficacy. Moreover, species selection affects removal capacity, and this study found *C. pseudocyperus* and *P. arundinacea* to be particularly efficient removers of Cu and Pb, and of Cd, Zn, and Cl^−^, respectively. These findings will be crucial when designing plant-based treatment systems in various environmental conditions.

## Data Availability

Full dataset will be made available upon request.

## Abbreviation

DW: Dry weight
